# Minimally invasive apical cannulation and cannula design for short-term mechanical circulatory support devices

**DOI:** 10.1186/s12872-022-02826-z

**Published:** 2022-09-04

**Authors:** Marcell Székely, Tamás Ruttkay, Ferenc Imre Suhai, Áron Bóna, Béla Merkely, László Székely

**Affiliations:** 1 Laboratory for Applied and Clinical Anatomy, Department of Anatomy, Histology, and Embryology, Semmelweis University, 58 Tűzoltó Street, Budapest, 1094 Hungary; 2 Heart and Vascular Center, Semmelweis University, 68 Városmajor Road, Budapest, 1122 Hungary; 3 Soós Research and Development Center, University of Pannonia, 18 Zrínyi Miklós Street, Nagykanizsa, 8800 Hungary; 4 Military Hospital Medical Centre, Cardiovascular and Thoracic Surgery Department, Hungarian Defense Forces, 44 Róbert Károly Boulevard, Budapest, 1134 Hungary

**Keywords:** Left ventricular assist device, Heart failure, Cardiogenic shock

## Abstract

**Background:**

Refractory cardiogenic shock is still a major clinical challenge with high mortality rates, although several devices can be used to conquer this event. These devices have different advantages and disadvantages originating from their insertion or cannulation method, therefore many complications can occur during their use. The aim of our study was to develop and create prototypes of a novel minimal invasively insertable, transapical cannula for surgical ventricular assist devices, which uniquely incorporates the inflow and outflow routes for the blood of the patient in itself, therefore it enables the use for only one cannula for patients in cardiogenic shock.

**Methods:**

To define the available space for the planned cannula in the left ventricle and ascending aorta, we analyzed computed tomography scans of 24 heart failure patients, who were indicated to left ventricular assist device therapy. Parallel to these measurements, hydrodynamical calculations were performed to determine the sizes of the cannulas, which were necessary to provide effective cardiac output.

**Results:**

After the designing steps, we produced prototypes of double-lumened, tube-in-tube apically insertable devices for three different patient groups, which included a separated venous and an arterial part using 3D modelling and printing technology. All the created cannulas are able to provide 5 l/min circulatory support.

**Conclusion:**

As a result of our research we created a sizing method based on the specific analysis of computed tomography pictures of end stage heart failure patients and a cannula concept, which can provide effective antegrade flow for patients in cardiogenic shock. We believe the improved version of our tool could have a significant therapeutic role in the future after further development based on animal and in vivo tests.

## Background

Cardiogenic shock (CS), even though processing surgical techniques, widespread use of primary percutaneous interventions, and medical treatment, remains a significant clinical challenge with high mortality rates [1]. There are several reasons responsible for this clinical event, for instance, acute myocardial infarction or acute manifestation of chronic heart failure.

In order to overcome the limitations and the failure of conventional treatment, to maintain adequate perfusion, and prevent irreversible end-organ failure, the use of short-term mechanical circulatory support (ST-MCS) has been significantly increasing in acute haemodynamic decompensation and refractory CS [2–4]. Some MCS devices should be implanted surgically, but there are some percutaneous systems like Impella, or veno-arterial extracorporeal membrane oxygenator (VA ECMO). These devices can ensure haemodynamic stability temporarily and they are also able to buy time for the regeneration of the myocardium [5]. There are several ideological indications to use the mentioned devices, such as bridge-to-transplant, bridge-to-decision, bridge-to-bridge if the weaning is impossible, and bridge-to-recovery. [2]

Two types of cannulation techniques exist between venous and arterial systems for these MCS devices. Arterio-arterial cannulation systems can function without an oxygenator and lack its disadvantages, such as plasma leak and bleeding [6, 7]. However, they usually require an invasive insertion and carry the negative consequences of it. Veno-arterial cannulation (VA-ECMO) is less invasive, but gas exchange is an essential part of the system, hereby it carries the previously mentioned complications caused by an oxygenator.

Using a percutaneous ST-MCS system for refractory CS, which can provide isolated left ventricular or biventricular circulatory support, has many advantages (quickness, minimal invasiveness) compared to surgical ventricular assist devices (VADs). However, they are limited in power, durability, and patient mobility after implantation, besides the high occurrence of different complications. [4, 6, 7]

Although the vast majority of ST-MCS devices are inserted percutaneously, for sufficient, durable (bridge-to-decision) circulatory support, surgical centrifugal pumps are often used in the therapy of acute CS [8]. The most common surgical approach for the implantation is median sternotomy, with or without a cardiopulmonary bypass (CPB), although it has several important drawbacks due to its invasiveness. [9]

The goal of our study was to develop a novel arterio-arterial cannula for surgical VADs, which could be implanted quickly and minimal invasively. We planned to create an apical two-in-one cannula gaining the possibility to combine a venous inflow (which is oxygenated arterial blood) and an arterial outflow tube in a single device. Using a tube-in-tube solution the inflow and outflow parts are separated completely from each other. The outer inflow tube ensures oxygenated blood flow from the cavity of the left ventricle (LV) to a centrifugal pump, then the inner longer outflow (arterial) tube guides the blood through the aortic valve into the aortic root above the openings of the coronary arteries. This system within the left heart provides systemic blood supply as well as increases coronary perfusion by receding blood from the ascending aorta.

We divided the development of the cannula into three steps. 1. We reconstructed the geometry of the LV and left ventricular outflow tract based on computed tomography (CT) scans of end-stage heart failure patients indicated for left ventricular assist device therapy. 2. We analyzed the radiological dimensions and made hydrodynamic calculations to determine the sizes of the cannulas. At this step, physical parameters of widely used hemodynamically stable cannula systems (Medtronic EOPA, LivaNova Dual Stage Venous Cannula) were taken as a sample. 3. We produced the prototypes of the cannula using 3D printing technology.

## Methods

### Measurements of the LV and LV outflow tract

All data acquisition and measurements were performed by an experienced radiologist during our anonymized retrospective analysis of previously made CT scans of 24 heart failure patients (EF < 20%). The patients belonged to INTERMACS III-IV classes with different etiologies of their heart failure. These etiologies contained ischemic cardiomyopathy (n = 10), dilated cardiomyopathy (n = 11), aortic regurgitation (n = 1), cardiac amyloidosis (n = 1), and muscular dystrophy (n = 1). Based on the status of their heart failure the patients were indicated to long-term left ventricular support therapy at the department of the third author from 2017 December to 2020 September. The CT scans of 19 men (79%), and 5 women were examined with the minimum age of 19, the maximal of 68, and the mean age of 51.52 years.

We performed retrospectively electrocardiogram-gated helical CT angiography (CTA) of the thorax to evaluate the thoracic anatomy before left ventricular support therapy. Images were acquired using a 256-slice CT scanner (Philips Healthcare, Best, The Netherlands, 270 ms rotation time, tube voltage of 100–120 kV, 128 × 0.625 mm collimation). We did not use oral or intravenous beta-blocker prior to CTA imaging. We administered 60 mL iodinated contrast agent (400 mg/mL, Iomeron 400, Bracco Ltd; Milan, Italy) with 4.5 mL/s flow rate and images were acquired with 1 mm slice thickness, 1 mm increment, and reconstructed using iterative reconstruction (iDose4 or IMR, Philips Healthcare, Cleveland, OH, USA). All image analyses were performed offline on dedicated cardiac workstations (Intellispace Portal, Philips Healthcare, Best, The Netherlands).

**Fig. 1:**
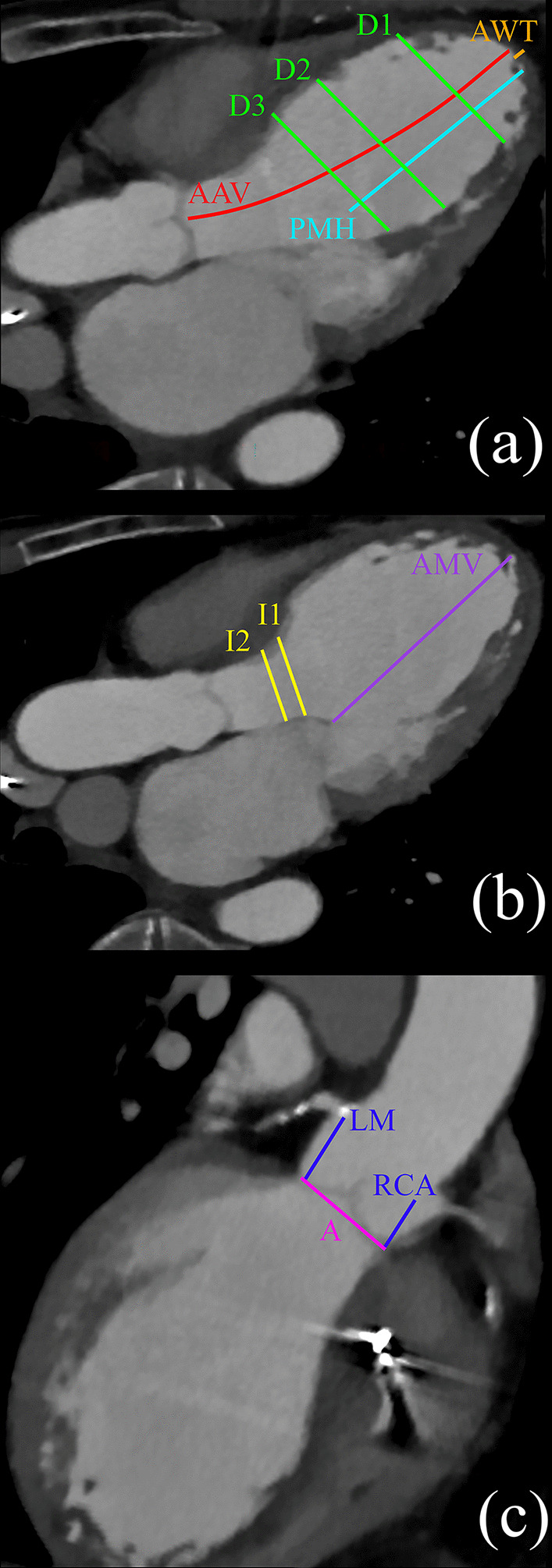
Studied cardiac CT images in diastole showing our measurement. In (**a**) and (**b**), the anterior mitral leaflet can be seen in an opened position. In (**c**), measures of the coronary artery openings and the annulus are visualized. The measured dimensions are seen with different colors and labeled appropriately with their abbreviation given detailed in the text

For the description of the left ventricular and left ventricular outflow tract geometry around the planned tube-in-tube cannula, we defined 14 measures in different planes of cardiac CT slides (Fig. 1). Measurements were performed in end-diastolic phase.

1. Apical wall thickness of the left ventricle at the cannula penetration point (AWT)
2. Inner diameters of the left ventricle in 3 planes between the interventricular septum and the anterior papillary muscle (D1, D2, D3).
3. Height of the anterior papillary muscle measured from the inner side of the apex (PMH).
4. Distance between the inner side of the apex and the margin of the anterior mitral leaflet in an opened position (AMV).
5. Distance between the inner side of the apex and the aortic valve annulus (AAV).
6. Inner diameters of the infundibulum in 2 planes (I1, I2).
7. Diameter of the left ventricular outflow tract (LVOT).
8. Diameter of the aortic valve annulus (A).
9. Distance between the aortic valve annulus and certain coronary artery openings (LM, RCA).
10. Angle enclosed by the axis of the apex-aortic valve annulus and the ascending aorta

### Analysis of the radiologic parameters and hydrodynamic calculations for the cannula design

The radiologic measures were necessary to specify the potential space for our double-lumened cannula in each patient from the apex to the aortic root. Before further analysis and determination of the cannula sizes, the patients were divided into three groups in accordance with their LV dimensions. Statistical analysis was performed using R 4.2.0. Continuous variables were reported as mean ± standard deviation and we determined the minimum and the maximum value of each measure in each group. We compared the mean values between the three groups regarding each measure using one-way analysis of variance (ANOVA), as our data followed normal distribution. A *p* < 0.05 was considered statistically significant.

To ensure the sufficient blood flow through the inflow and outflow parts of our transapical cannula, we took the inner diameter (12 mm venous, 8 mm arterial) of widely used cannulas from cardiac surgical practice. We designed a double-lumened device, which has hydrodynamically equivalent tubes, and which is potentially able to provide a 5 l/min cardiac output, producing a similar pressure drop. Reynolds numbers were calculated to compare the flow regime in the novel double-lumened cannula to the typical products used today.

Regarding the construction of the physical parameters of the cannula, we had to decide the length and the width both for the outflow and inflow tubes. Furthermore, we assigned the appropriate position, amount, and size of the inlets on the outer venous tube.

### 3D modelling and printing of the designed cannula

**Fig. 2:**
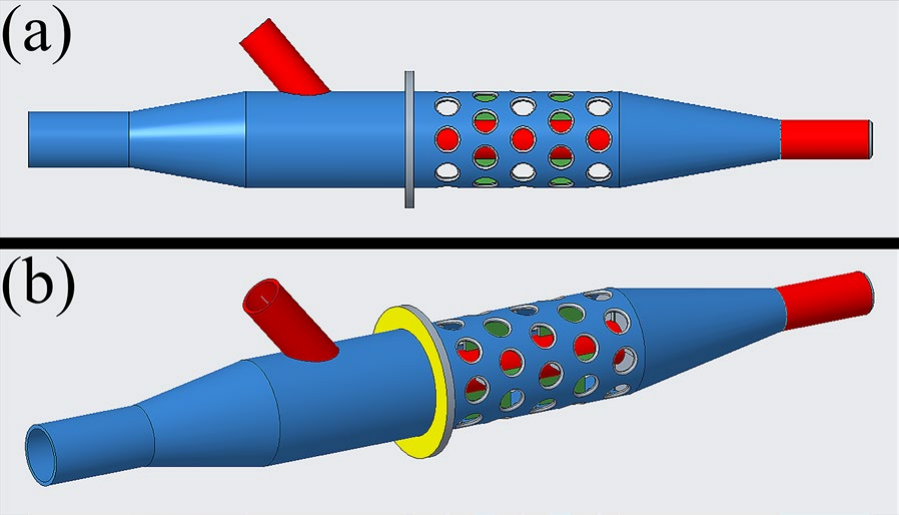
Two renders of the double-lumened cannula (middle size) in the used modelling software in different views: **a** top view, **b** perspective view. The venous tube is marked in blue, and the arterial tube is marked in red. The yellow circular skirt was made to mark and hold the outer side of the apical wall. The spacers between the venous and arterial tubes are marked in green

**Fig. 3:**
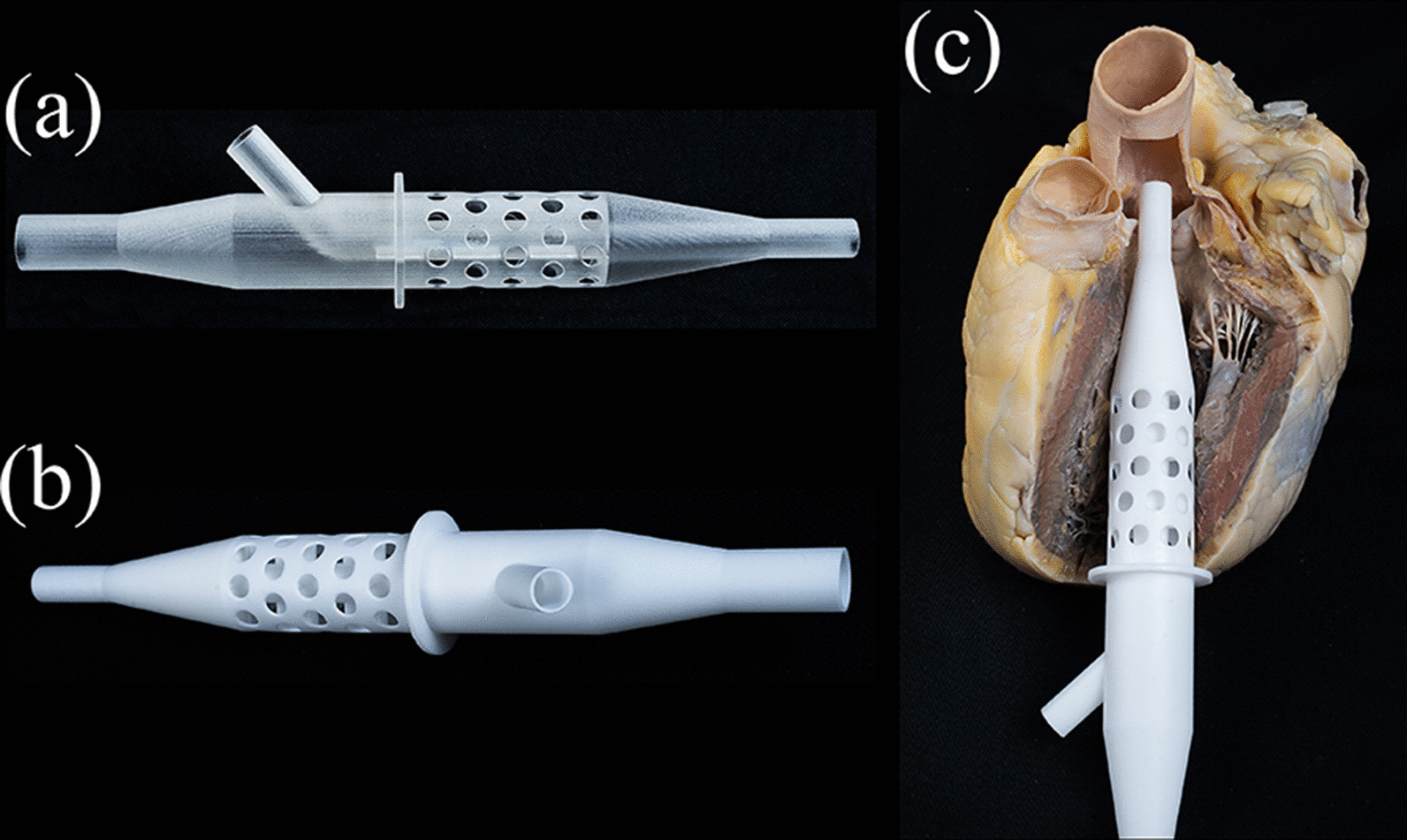
Photographs of our 3D printed cannula prototypes. **a** Model printed from UltraVeroClear; **b** Model printed from PA2200. **c** Double-lumened cannula placed in a prepared, fixed human heart to depict the anatomical and optimal position of the cannula

We constructed our cannulas (Fig. 2a, b) in PTC Creo software (PTC, Boston, USA) used for 3D modelling and printing. UltraVeroClear material and Polyjet J750 printer (Stratasys, Rehovot, Israel) were used for one prototype (Fig. 3a) and PA2200 material in an EOS P396 printer (EOS GMBH, Krailling, Germany) for the other (Fig. 3b). The printed models and their presented position in a prepared fixed human heart were documented with a colored photograph (Fig. 3c).

## Results

The three groups of the hearts were divided based on their AAV value as it was the most critical and the most variable measure. The minimum of the AAV was 91.2 mm and the maximum was 122.6 mm. We separated 3 equal parts between these values (31.4 mm interval). The created 3 parts were named: 91.2–101.66 mm as group 1, 101.66–112.12 mm as group 2 and 112.12–122.6 mm as group 3. Group 1 contained 5, group 2 contained 7, and group 3 held 12 patients. Further on describing each size, we used this classification.

### The length of the arterial (outflow) tube within the left ventricle and aortic root

Regarding our theory, the cannula optimally starts from the outer side of the apical wall and ends above the openings of the coronary arteries. To define this parameter of the cannula in each size, we summated the maximum values of AWT, AAV, and LM or RCA in each group. Examining the heights of LM and RCA, we took only the larger values into notice. In each group, 5 mm correction was added to coronary heights in order to leave enough space for the receding blood to the coronary arteries from the top of the cannula. Regarding our examination, we defined the full length of the cannulas in each size as 129.76 mm in group 1, 139.12 mm in group 2, and 152.80 mm in group 3. (Table 1).

**Table 1:** Important measures regarding the length of the arterial tube of the different cannula sizes

	Group 1	Group 2	Group 3	*p* value
Patients number	5	7	12	
AWT (mm) min–max	2.2–5	1.5–4.3	2.3–4.2	0.205
Mean ± SD	3.82 ± 1.19	2.84 ± 1.06	3.24 ± 0.65	
AAV (mm) min–max	91.2–101.66	101.66–112.12	112.12–122.6	< 0.0001
Mean ± SD	97.52 ± 4.35	105.86 ± 3.52	116.82 ± 3.19	
LM (mm) min–max	10.3–14.5	10.7–14.6	10.7–15.8	0.649
Mean ± SD	12.84 ± 1.73	12.84 ± 1.39	13.47 ± 1.71	
RCA (mm) min–max	12–18.1	14.6–17.7	10.3–21	0.466
Mean ± SD	14.32 ± 2.48	16.04 ± 1.26	15.88 ± 3.08	
Sum of the max. values (mm)	124.76	134.12	147.8	
Sum after the 5 mm correction (mm)	129.76	139.12	152.8	

*AWT* apical wall thickness, *AAV* distance between the inner side of the apex and the aortic valve annulus, *LM* distance between the aortic valve annulus and the left main coronary artery opening, *RCA* distance between the aortic valve annulus and the right coronary opening, *SD* standard deviation; Continuous variables are reported as mean and standard deviation; *p* < 0.05 indicates statistical significance

### Length of the double-lumened venous (inflow) part of the cannula

Between the outer side of the apical wall and the margin of the anterior mitral leaflet in an opened position, the venous tube can be designed around the arterial tube without damaging the tendinous chords. This is the double-lumened part. This size was defined with the summation of the maximum value of AWT and the minimum value of AMV in each heart group. (Table 2).

**Table 2:** Significant measures for defining the length of the double-lumened part of the cannulas

	Group 1	Group 2	Group 3	*p* value
Patients number	5	7	12	
AMV (mm) min–max	64.6–74	64.1–77.6	66.6–86	0.023
Mean ± SD	68.68 ± 3.52	70.71 ± 5.12	76.63 ± 6.45	
Minimum of AMV (mm)	64.6	64.1	66.6	
Maximum of AWT (mm)	5	4.3	4.2	
Sum maximum of AWT and minimum of AMV (mm)	69.6	68.4	70.8	

*AMV* distance between the inner side of the apex and the margin of the anterior mitral leaflet in an opened position, *AWT* apical wall thickness of the left ventricle at the cannula penetration point, *SD* standard deviation; Continuous variables are reported as mean and standard deviation; *p* < 0.05 indicates statistical significance

In the length of the double-lumened part, the outer tube wall contains inlets for the inflow of blood. At the endpoint of this venous part, the wall runs conically into the wall of the arterial tube to avoid apical myocardial damage by the insertion of the cannula. This conically ending wall was designed to end between the margin of the anterior mitral leaflet and the aortic valve in each patient group. We reduced the length of the double-lumened part to 51 mm in all prototypes from the measured longer distances, because the elongation of this conical closure of the outer cannula was necessary to the safe insertion.

### Width of the venous and arterial tubes (outflow and inflow) of the cannula

First, we described the available space for the double-lumened, thicker part of the cannula using the minimum values of the D2 and D3 in each group. (Table 3) Then, to assess the space for the thinner arterial tube, we examined the I1, I2, LVOT and Annulus measures in each group. From all these four measures, we only accounted the minimum value, to estimate the maximal space for the tube without hurting any structures. (Table 4).

**Table 3:** Measures for defining the width of the venous part of the cannulas

	Group 1	Group 2	Group 3	*p* value
Patients number	5	7	12	
D2 (mm) min–max	36.2–49.8	29–59.4	35–63.2	0.043
Mean ± SD	42.44 ± 6.59	43.76 ± 9.17	51.98 ± 7.72	
D3 (mm) min–max	29.8–49	24.1–45.7	29.8–60	0.154
Mean ± SD	37.72 ± 7.29	34.9 ± 9.04	43 ± 8.99	
Minimum of previous diameters (mm)	29.8	24.1	29.8	

*D2, D3* inner diameters of the left ventricle between the interventricular septum and the anterior papillary muscle,*SD* standard deviation; Continuous variables are reported as mean and standard deviation; *p* < 0.05 indicates statistical significance

**Table 4:** Critical diameters for assessing the space in the path of the arterial tube

	Group 1	Group 2	Group 3	*p* value
I1 (mm) min–max	18.8–27.5	21.3–32.1	21–31.7	0.069
Mean ± SD	23.02 ± 3.08	26.07 ± 3.62	27.3 ± 3.13	
I2 (mm) min–max	20.2–32	26.7–41.3	24–46.5	0.018
Mean ± SD	26.32 ± 5.31	32.73 ± 5.12	35.7 ± 5.98	
LVOT (mm) min–max	16–35.7	19.8–38.1	22–38.3	0.236
Mean ± SD	25.96 ± 5.78	28.21 ± 5.81	29.76 ± 5.98	
Annulus (mm) min–max	18–32.2	19.7–34.2	22.7–34.6	0.075
Mean ± SD	24.82 ± 3.99	26.39 ± 4.39	28.26 ± 3.91	
Minimum of previous diameters (mm)	16	19.7	21	

*I1, I2* inner diameters of the infundibulum in 2 planes, *LVOT* diameter of the left ventricular outflow tract, Annulus: Diameter of the aortic valve annulus, *SD* standard deviation, Continuous variables are reported as mean and standard deviation; *p* < 0.05 indicates statistical significance

Besides the definition of the available space in the left heart, hydrodynamic calculations were needed to decide, whether this space is sufficient for our goal.

We based our calculations on 5 l/min flow rates. The inner (arterial) tube was decided to have an inner diameter (ID) of 8 mm in all prototypes. The outer, annular tube was designed to have at least the same hydraulic diameter as a commercially available venous cannula with a 12 mm ID, which in our case leads to an annular slit width of 6 mm. To partially compensate for the loss of cross-section area due to the three spacers connection between the inner and outer tubes, we raised the slit width to be 6.20 mm, leading to an overall 24 mm outer diameter for our double-lumened cannula. We used that measure in all sizes as the width of our cannulas.

Both venous and arterial cannulas are designed to have Reynolds numbers in the high transitional, lower turbulent flow regions. The Reynolds numbers were calculated for both the 12 mm ID venous cannula and the venous (annular) part of the double-cannula, showing that the annular flow has a Reynolds number of approx. 2000, compared to the approx. 5000 of the 12 mm tubular cannula. A 2000 Reynolds number is on the laminar/transitional limit, which might cause some concern regarding blood clotting on the cannula surface. However, the danger of clotting can be mitigated by creating a non-adhesive surface, and by optimal anticoagulant medication [10]. Furthermore, by designing the spacer to act as a turbulence promoter, the turbulence of the flow can be increased considerably. To address the exact hemodynamic flow conditions within the double-lumened cannula, numerical simulations are needed, which we plan to carry out in the near future.

### Design of the parameters of the venous tube

For this part of the study, we determined the amount, the size, and the appropriate place for the inlets on the venous tube. To construct these parameters, we considered hydrodynamical calculations, anatomical, and hemodynamic factors.

A 5.40 mm hole size was chosen for the sucking inlets, with 4.00 mm spaces between the holes to provide stability for the outer wall. This leads to 8 holes circularly around the outer circle of the wall of the cannula. To provide enough space for blood flow, even if the cannula is close to the inner wall of the heart, we doubled the cross-section area of the annular cannula to calculate the minimal area for the sum of the inlets, which leads to 3.3 inlet rows. Between each inlet row, we left a 4.00 mm distance to keep the stability of the cannula. Therefore, according to our calculations, with these parameters, only 4 inlet rows on the outer tube would be plenty to forward the 5 L/ minute cardiac output from the LV towards the extracorporeal pump, however, we found it more beneficial to place 5 inlet rows until the length of the outer tube in each cannula models. Until this point, the inlets cannot damage the mitral valves and cause any following adverse event by it. The lowest inlet row on the cannulas starts 1.50 mm above the maximum value of AWT from the outer side of the apex for each group.

The estimated outer side of the apical wall was marked on our cannulas with a circular skirt. Outwards the skirt, the cannula parts were designed to be eligible for further experiments. (Figs. 2a, b, 3a, b).

## Discussion

Acute cardiogenic shock has always been a significant problem in hospitals due to its high mortality [11]. There are several devices available to overcome this life-threatening situation, but each device still has its own limitations regarding flow capability, requirement of special skill for insertion, ventricular unloading effect and durability, among many others. [4, 5]

Firstly, we will discuss the reasons behind our decisions to develop a new cannula conception. Secondly, the advantages of this cannula will be presented comparing to other existing ST-MCS devices (VA ECMO, Impella, Surgical VAD). Finally, we will detail the limitations of our study and tool.

Since all existing devices have limitations, we planned and managed to create a hemodynamically functioning double-lumened cannula to produce a different tool to conquer refractory CS. The cannula would operate as a part of an extracorporeal continuous-flow VAD, connected to a centrifugal pump, inserted from a minimally invasive left anterolateral thoracotomy, through the apex. If biventricular support is necessary, it can operate with an oxygenator, but for LVAD purpose, it is considered a strength that an oxygenator is not needed in the system.

The construction of the cannula was based on hydrodynamical calculations and the analysis of radiological images of the previously described patient population, who would be potentially indicated for the insertion of our cannula.

With the help of the radiological dimensions, we assessed the possible maximal space for our model cannulas from the apex of the LV to the aortic root. These measures were important to describe the dimensions of our double-lumened cannula, without damaging any structures of the heart, while supporting heart failure patients with sufficient cardiac output. The aimed and reached capacity for our device was to ensure a 5 L/minute cardiac output.

We separated the hearts into three groups to create three different sizes for our cannula models, because of the significant variability of radiological measures. A too long cannula would satisfy systemic blood demand but would not be able to ensure proper coronary perfusion. On the other hand, a too short cannula would not reach the coronary artery openings or the aortic valve, therefore the systemic and coronary blood supply would not be sufficient. Producing more sizes of the device, an optimal cannula can be selected for each patient, however with our measuring method and 3D technology, cannulas can even be personalized for them.

As a complement to our results regarding the available left ventricular inner dimensions, hydrodynamic calculations were done based on the parameters of routinely used parts of CPB equipment. A 24 French (8 mm) arterial cannula (Medtronic EOPA) can safely ensure the necessary cardiac output during CPB, hereby we decided to create the arterial tube of our 3D printed cannulas with the same ID. Moreover, our cannula’s venous capacity is similar to a 36 French (12 mm) venous cannula (LivaNova) to guarantee sufficient flow.

The number and position of the inlet rows on the outer tube were also important questions of our study. We decided to place the first inlet row as near to the apical wall as it was possible, however surely above the inner side of the apical wall. It was necessary to avoid potential thrombus formation between the first inlet row and the apical wall to prevent concomitant complications. Based on our hydrodynamic calculations, fewer inlet rows were able to ensure an adequate flow, but due to suction, numerous inlets could be closed through sticking of the endocardium of the papillary muscle or ventricular wall. We found no contraindications interrelated to more inlet rows, therefore we opted for a wide safety margin.

### ECMO

VA ECMO insertion has been increasing in the past few years, as it has an absolute indication in refractory CS, and in several other cases [12]. It is able to provide circulatory and respiratory support at the same time. As in VA ECMO, a long venous cannula is placed in large central veins, inserted into the right atrium, where venous blood is drained into the circuit. The blood undergoes gas exchange in a polymethylpentene hollow-fiber membrane oxygenator, and is pumped back to the systemic circulation via a shorter arterial cannula towards the aorta by a centrifugal pump. [12]

Despite ECMO’s widespread usage in adult CS, survival to discharge is less than 45% [13]. No other ST-MCS device has better results in these cases compared to VA ECMO, however, the low percentages of survival occur due to several complications of ECMO. [6]

One of the most important characteristics of ECMO’s veno-arterial cannulation is the watershed phenomenon. It is the consequence of retrograde blood flow delivered by the ECMO unit and the antegrade flow produced by the failing heart. Their meeting point is called watershed [12, 14]. The location of the watershed is determined by the scale between the retrograde and the antegrade flow [15]. The antegrade blood flow coming from the LV is oxygenated by the patient’s pulmonary gas exchange system. If any damage in oxygenation occurs, insufficiently oxygenated blood would perfuse areas proximal to the watershed, which can result in hypoxic damage of the heart and the brain, despite good perfusion pressure. The main problem, in this case, is that blood gases obtained from the right radial artery may not reflect the oxygen content of the blood delivered to the coronary arteries. [14]

Another significant problem connected to retrograde blood flow coming from the arterial cannula of VA ECMO is the distension of the LV. Retrograde blood flow increases afterload of the heart and ejection is diminished. The blood coming from the thebesian and bronchial veins distends the LV and facilitates sludge development [12]. Consequently, these effects increase myocardial oxygen requirement, hereby increasing the chance of peripheral or central embolization, worsening LV recovery and the outcome of the patients. [16]

Our cannula connected to an extracorporeal centrifugal pump system unloads the LV by the inflow tube and generates only an antegrade flow by the outflow tube. Accordingly, the watershed phenomenon, and its negative consequences, for instance, neurological hypoxaemia, myocardial distension, thrombus formation and embolization can be avoided by this technique.

Secondly, the use of femoral arterial cannulation in a VA ECMO system reduces perfusion distal to punction site, hereby lower limb ischaemia can potentially occur. Therefore, the patient may need vascular surgery, or in critical situation, amputation [6, 17]. Additionally, this cannulation involves the chance of arterial injury. Obviously, the presence of vascular complications and lower limb ischaemia would not likely show up with the use of our cannula, since this technique does not require arterial punction and femoral cannulation.

Furthermore, the most frequent complication, related to VA ECMO therapy in adult patients is bleeding. The presence of this adverse event in most cases is associated with the use of a membrane oxygenator, because blood is exposed to artificial surfaces and high shear from centrifugal flow pumps. When an oxygenator takes part in the circulatory support system, coagulation factors become deteriorated and the coagulation system suffers several damages, hereby it appears, for instance as bleeding with increased transfusion requirement. Additionally, if the previously mentioned complication does not show up, bleeding is henceforward a usual danger, because of the massive heparinization of the patient in order to prevent thrombotic events and oxygenator thrombosis, while using an ECMO circuit [18, 19]. This type of heparinization differs from the heparinization, for example a surgical VAD needs, because a support system with an oxygenator requires a higher level of anticoagulation therapy than a system, which lacks this accessory [20]. Without the use of an oxygenator, whether it is possible, the risk of bleeding becomes considerably lower. Our cannula would operate by oxygenated blood from the LV without the need of an oxygenator, and its notable bleeding risks.

### Impella

Another percutaneous ST-MCS device is the Impella microaxial pump. It provides circulatory support similar to VADs, although it has the advantage, that it can be inserted minimal invasively, because its miniaturized size [5]. This is the only device for acute CS therapy, which generates antegrade blood flow alone, as it is positioned across the aortic valve to provide transvalvular LV assistance. There are several versions available from Impella devices [4]. The smaller sizes are favorable as they cause less damage to the vascular system of the patient due to its percutaneous insertion and working period. However, the capacity to generate antegrade blood flow is narrower compared to bigger sizes, whose insertion requires surgical exploration [21]. As a result of LV unloading by the pump, it reduces left ventricular end-diastolic pressure, LV wall stress, and myocardial oxygen demand. Blood flow delivered by Impella improves cardiac index, mean arterial pressure, coronary flow, and end-organ perfusion.

The mentioned positive effects of this device are reasonably promising, although, Impella also has several disadvantages. Limitations include the high rotational speed of the axial flow pump with consecutive haemolysis, the high risk of femoral bleeding and limb ischaemia, vascular complications, and the absence of improved pulmonary oxygenation [4]. There is only a limited number of publications, proving favorable outcomes using Impella, compared to other ST-MCS devices. [21]

We consider our apically insertable cannula with extracorporeal pump system a potentially favorable choice over the Impella, because limitations in capacity, high rotational speed caused heamolysis, and limb ischaemia would not occur using our technique, since the motor size, as in Impella, is not a component, which has any limitation.

### Surgical VADs

Along with many percutaneously inserted ST-MCS devices, there is the possibility to use temporary (day to weeks), surgical VADs for the treatment of patients in acute, refractory CS. For advanced heart failure patients, they are available for the indication of bridge-to-transplant, and bridge-to-recovery [8]. Centrifugal pumps possess the advantages of versatility, easiness of use, and relatively low cost. The Centrimag device can provide circulatory support for 30 days, hereby its use has been spread out more during the years, than other pumps [22]. One of the main disadvantages concerning this device, that the conventional surgical access for implantation is median sternotomy, with or without a CPB [9]. Because of this invasive cannulation method several complications can occur, such as bleeding, infection, increased time on a CPB, requirement of right ventricular assist and transfusion. [23]

To reduce the presence of these complications, numerous minimally invasive techniques have been evaluated in the past years, regarding the insertion of surgical VADs [23–27]. In a retrospective review, where several continuous flow LVAD implantations were examined, less minimally invasive cases required more right ventricular assist than median sternotomy cases [23]. In another comparison according to Centrimag insertion, less amount of blood transfusion and a significantly lower incidence of major bleeding events were found during support [25]. Several more beneficial outcomes of minimally invasive techniques can be seen by these examples, besides sparing the sternum of the patient for a potential subsequent definitive surgery.

All mentioned special methods for the implantation of surgical VADs, even the less invasive technique, require minimum two incisions on the thorax. One for the insertion of the inflow cannula, and one for the outflow cannula. Contrarily, the insertion of our cannula would only need one small incision above the apex, using a left anterolateral minithoracotomy. With this technique, we would reduce the time of surgery and decrease wound surface.

### Limitations

We performed detailed radiologic measurements and hydrodynamic calculations to create our double-lumened cannula models, but the in vivo proofing of the system, and animal studies have not been executed yet. Furthermore the optimal material of the cannulas, which should ensure the appropriate elasticity and biocompatibility for the tools has not been finalized as well. Finally, further studies will be required to prove the potential advantages and show the possible complications after implantation.

## Conclusions

The aim of our study was to design prototypes of a different cannulation and cannula concept for short-term left ventricular assist device therapy. With this novel cannula concept, we wanted to develop a device, which can be connected to centrifugal pumps, and can provide circulatory support with only antegrade flow. The cannulas can be inserted transapically, via left anterolateral minithoracotomy. We analyzed CTA scans of end stage heart failure patients to assess the space in their LV and aortic root. After the performed radiologic measurements and hydrodynamic calculations we developed and printed our double-lumened cannula models with 3D printing technology in three different sizes for three different patient groups. Important requirements were for the products, that they should be placed in the patients without the potential damage in any structures, and they should ensure sufficient circulatory support (5 l /min). We concentrated on the sizes and form of the tool. As the next step, it will be an important question to find the optimal material for the cannula and perform animal studies to find out possible complications regarding our novel device. In addition, a radiological method to certificate the adequate position of the cannula after implantation should be worked out as well.

## Data Availability

The datasets used and/or analysed during the current study are available from the corresponding author on reasonable request.

## Abbreviations

CS: Cardiogenic shock
ST-MCS: Short-term mechanical circulatory support
MCS: Mechanical circulatory support
VA ECMO: Veno-arterial extracorporeal membrane oxygenation
VAD: Ventricular assist device
LVAD: Left ventricular assist device
CPB: Cardiopulmonary bypass
LV: Left ventricle
LVOT: Left ventricular outflow tract
ID: Inner diameter
EF: Ejection fraction
INTERMACS: Interagency Registry for Mechanically Assisted Circulatory Support
CT: Computed tomography
CTA: Computed tomography angiography
